# The Gd_2−x_Mg_x_Zr_2_O_7−x/2_ Solid Solution: Ionic Conductivity and Chemical Stability in the Melt of LiCl-Li_2_O

**DOI:** 10.3390/ma15124079

**Published:** 2022-06-08

**Authors:** Irina Anokhina, Olga Pavlenko, Natal’ya Proskurnina, Alexander Dedyukhin, Irina Animitsa

**Affiliations:** 1 The Institute of High Temperature Electrochemistry of the Ural Branch of the Russian Academy of Sciences, 620990 Yekaterinburg, Russia; o.pavlenko@ihte.uran.ru (O.P.); dedyukhin@ihte.uran.ru (A.D.); 2 The Institute of Metal Physics of the Ural Branch of the Russian Academy of Sciences, 620108 Yekaterinburg, Russia; proskurnina@imp.uran.ru; 3 Institute of Natural Sciences and Mathematics, Ural Federal University, 620002 Yekaterinburg, Russia

**Keywords:** Gd_2_Zr_2_O_7_, pyrochlore structure, oxygen vacancies, oxygen-ion conductivity, chemical stability in the halide melt

## Abstract

Materials with pyrochlore structure A_2_B_2_O_7_ have attracted considerable attention owing to their various applications as catalysts, sensors, electrolytes, electrodes, and magnets due to the unique crystal structure and thermal stability. At the same time, the possibility of using such materials for electrochemical applications in salt melts has not been studied. This paper presents the new results of obtaining high-density Mg^2+^-doped ceramics based on Gd_2_Zr_2_O_7_ with pyrochlore structure and comprehensive investigation of the electrical properties and chemical stability in a lithium chloride melt with additives of various concentrations of lithium oxide, performed for the first time. The solid solution of Gd_2−x_Mg_x_Zr_2_O_7−x/2_ (0 ≤ x ≤ 0.10) with the pyrochlore structure was obtained by mechanically milling stoichiometric mixtures of the corresponding oxides, followed by annealing at 1500 °C. The lattice parameter changed non-linearly as a result of different mechanisms of Mg^2+^ incorporation into the Gd_2_Zr_2_O_7_ structure. At low dopant concentrations (x ≤ 0.03) some interstitial positions can be substituted by Mg^2+^, with further increasing Mg^2+^-content, the decrease in the lattice parameter occurred due to the substitution of host-ion sites with smaller dopant-ion. High-density ceramics 99% was prepared at T = 1500 °C. According to the results of the measurements of electrical conductivity as a function of oxygen partial pressure, all investigated samples were characterized by the dominant ionic type of conductivity over a wide range of pO_2_ (1 × 10^–18^ ≤ pO_2_ ≤ 0.21 atm) and T < 800 °C. The sample with the composition of x = 0.03 had the highest oxygen-ion conductivity (10^−3^ S·cm^−1^ at 600 °C). The investigation of chemical stability of ceramics in the melt of LiCl with 2.5 mas.% Li_2_O showed that the sample did not react with the melt during the exposed time of one week at the temperature of 650 °C. This result makes it possible to use these materials as oxygen activity sensors in halide melts.

## 1. Introduction

The pyrochlore oxides with the general formula of A_2_^3+^B_2_^4+^O_7_, where A^3+^ is a rare-earth ion and B^4+^ is usually a transition metal, have attracted great attention as materials for nuclear waste disposal [1,2,3], thermal barrier coating materials [4,5,6], catalysts [7,8], and for other important technological applications such as solid electrolytes [9,10], electrodes [11], and magnets [12,13,14].

The ideal stoichiometry of pyrochlore structure is A_2_B_2_O_6_O′. The structure contains crystallographic unoccupied anion sites: six oxygen atoms occupy the 48*f* sites, surrounded by two A cations and two B cations, and the seventh oxygen atom occupies the 8*b* site and is surrounded by four A cations; the remaining unoccupied 8*a* site is surrounded by B cations [15]. So, the anion vacancies are ordered in the ideal pyrochlore structure. Such structural features of pyrochlore create the possibility of anti-Frenkel disordering: O_o_^×^ ↔V_o_¨(48*f*) + O*_i_*″(8*b*) [16]. Consequently, the existence of intrinsic anion vacancies suggests, that pyrochlore compounds can exhibit fast oxygen ion conductivity. In this regard, in recent years, pyrochlores have been intensively studied as oxygen-ion conductors for the using such systems in electrochemical applications [11]. So, an important task is the development of materials with high oxygen-ion conductivity, stable over a wide range of temperatures and oxygen partial pressures. One of the strategies for improving these properties is the method of acceptor doping. The doping method is expected to introduce such defects as oxygen vacancies and change the lattice energy of the crystal. Among compounds with pyrochlore structure, the highest values of oxygen ion conductivity were realized for doped gadolinium zirconate Gd_2_Zr_2_O_7_. Numerous studies have described various types of cationic substitutions [17,18,19,20,21,22,23,24,25,26,27,28,29]. At the same time, from the various types of cation substitutions, magnesium, as a dopant, has advantages. Introduction of Mg^2+^ makes it possible not only to increase the ionic conductivity, but also to significantly improve the sintering process. The possibility of obtaining high-density ceramics is an important requirement for the electrochemical applications. The using of magnesium as a dopant for the compounds with the pyrochlore structure has been reported in the several studies [30,31,32,33,34], although the Mg^2+^-substituted solid solution based on Gd_2_Zr_2_O_7_ has not been studied yet.

The solid solution Sm_2−x_Mg_x_Zr_2_O_7−x/2_ [30] was prepared by a solid-state reaction and characterized by X-ray diffraction, high-temperature dilatometry, Raman spectroscopy and X-ray photoelectron spectroscopy. It is interesting to note that a new solid-solution mechanism was proposed and confirmed by analyzing the variation of the lattice parameters, experimental density and X-ray photoelectron spectroscopy of the samples. The authors suggested that at low concentrations of magnesium, interstitial magnesium is formed Mg*_i_*¨. With a further increase in the dopant concentration, the usual substitution mechanism is realized with the formation of the corresponding defects Mg′_Ln_ (divalent Mg cation at a trivalent Ln cation site) and V_o_¨ (an oxygen vacancy). The main focus of this work was to determine the linear thermal expansion coefficients due to the potential applications of such materials as thermal barrier coatings. Later, Mg^2+^-doped pyrochlores have attracted attention in terms of their transport properties.

The solid solution GdSm_1−x_Mg_x_Zr_2_O_7−x/2_ was investigated by Z.-G. Liu et al. [31]. It was shown that the samples were oxide-ion conductors, and that total conductivity of GdSm_1−x_Mg_x_Zr_2_O_7−x/2_ ceramics slightly decreased with increasing magnesia content, but at temperatures of 1123–1173 K the total conductivity slightly increased. It is also important to note that Mg^2+^-doping promoted the sintering densification behavior [31].

Yusen Wu et al. [32] have reported that the introduction of Mg^2+^ in La^3+^-sublattice of La_2_Ce_2_O_7_ led to an increase in conductivity and an improvement in the sintering of ceramics. Moreover, the samples showed excellent chemical stability under wet CO_2_.

The composition Gd_1.9_Mg_0.1_Zr_2_O_7−x/2_ was synthesized by Shlyakhtina A.V. et al. [33]: the sample had pyrochlore structure, but the electrical properties have not been studied.

Mg^2+^-doped Sm and Gd zirconates with composition Sm_1.9_Mg_0.1_Zr_2_O_6.95_ and Gd_1.9_Mg_0.1_Zr_2_O_6.95_ were studied by V. Sadykov et al. [34] in terms of their diffusion properties as promising materials for solid oxide fuel cells (SOFCs). It has been proven that the materials are oxide ionic conductors.

Summarizing the results of these works, we can conclude that Mg^2+^-doping is an effective strategy for improving both sintering and electrical conductivity. In this work, we decided to extend this approach to pyrochlore with the composition of Gd_2_Zr_2_O_7_. The electrical properties of Mg^2+^-substituted solid solutions based on Gd_2_Zr_2_O_7_ have not been previously studied.

It is important to emphasize, that it has recently been shown that gadolinium zirconate Gd_2_Zr_2_O_7_ with the pyrochlore structure has great prospects for application as an oxygen sensor for salt melts [35]. The application of solid-state electrolytes as gas sensors or sensors in the solution media is widely developed. However, the investigation of solid electrolyte type sensors for molten salts is rarely reported. The development of such sensors is an important task in the field of pyrochemical reprocessing of spent nuclear fuel and for long-term storage or final disposal of high-level nuclear wastes. Monitoring of the Li_2_O concentration in molten LiCl-Li_2_O electrolyte fuels is necessary for optimum operation and corrosion mitigation of the platinum anode materials. The literature on this field is extremely limited. Several works describe the corrosion behavior of ceramic materials based on the yttria stabilized zirconia electrolyte [36,37,38,39,40,41], but the corrosion instability of ZrO_2_–based ceramic electrolytes in Li^+^—containing melts was found. Therefore, the search for the new materials with complex properties such as high oxygen-ion conductivity and corrosion resistance in halide melts is extremely important. As follows from the work [35], the pyrochlore Gd_2_Zr_2_O_7_ is a promising compound for its further modification and investigation in the molten salts.

Therefore, in addition to investigation of the oxygen-ion conductivity of the solid solution Gd_2−x_Mg_x_Zr_2_O_7−x/2_, another goal of this work is to undertake studies of the obtained phases in terms of their chemical stability in the LiCl-Li_2_O melt with the aim of further selection of promising compositions for using as oxygen activity sensors for the salt melts.

In this study, Gd_2−x_Mg_x_Zr_2_O_7−x/2_ ceramics were prepared, their conductivities over a wide range of the temperatures (300–1000 °C) and partial pressures of oxygen (1 × 10^–18^–0.21 atm) were investigated for the first time. The effect of MgO-doping on the electrical properties and chemical stability of Gd_2_Zr_2_O_7_ in the LiCl–Li_2_O melt were investigated for the first time.

## 2. Materials and Methods

### 2.1. Sample Preparation

The samples Gd_2−x_Mg_x_Zr_2_O_7−x/2_ (x = 0, 0.03, 0.04, 0.05, 0.10, 0.15, 0.20) were prepared by mechanical milling starting reagents Gd_2_O_3_ (99.998%, VEKTON, St. Petersburg, Russia, RF), ZrO_2_ (99.99%, REACHIM, Moscow, Russia), and MgCO_3_ (99.99%, VEKTON, St. Petersburg, Russia), taken in stoichiometric amounts and followed by high-temperature treatment at 1500 °C for 5 h. Before high-temperature treatment the mechanical activation of the mixture was made in a planetary ball mill (Fritsch Planetar Micro Mill PULVERISETTE 7, Markt Einersheim, Germany) by using 125-mL zirconia container with 5 mm zirconium balls at a speed of 350 rpm for 8 h in ethanol. The ball-to-powder mass ratio was 10:1. After the milling, the mixture was dried at 100 °C, pressed into pellets on a hydraulic press (Lab tools, RF) at ~1 MPa and sintered at 1500 °C for 5 h. The heating and cooling rate was 1°/min.

### 2.2. Structural Characterization

The crystal structure of the prepared samples was characterized by an X-ray diffractometer (Rigaku MiniFlex600, Akishima-shi, Japan) with Cu-Kα radiation at a scanning rate of 0.5°/min and step of 0.01° using the SmartLab Studio II software (Version 4.3.200.0, Akishima-shi, Japan) and the PDF 2–2019 database. The silicon powder NIST640f (Gaithersburg, MD, USA) was used as the external standard. Refinement of the structural parameters was carried out by the Rietveld method using the Full Prof program (Version April 2019, Grenoble, France).

### 2.3. Microstructural Characterization

The microstructure of the sintered specimens was investigated by a scanning electron microscope (TESCAN MIRA 3 LMU, Brno, Czech Republic). The specimens were polished on a diamond wheel, and a finely dispersed layer of copper was deposited on the surface of the ceramic pellets. Thereafter, the surfaces of the pellets were analyzed.

### 2.4. Chemical Composition Analysis

Chemical analysis of Gd^3+^, Zr^4+^, and Mg^2+^ was carried out by inductively coupled plasma atomic emission spectroscopy on an Optima 4300 DV Perkin Elmer spectrometer (Waltham, MA, USA); the oxygen content was calculated according to the cationic composition. Preliminarily, the ceramic samples were ground and then dissolved in a mixture of sulfuric acid and ammonium sulfate in the ratio of 3:2 by weight.

Energy dispersive X-ray spectroscopy (EDS) in the SEM was used to analyze the composition of pelletized samples. Samples were examined by using INCA Energy 350 + XEDS detector, based an Oxford Instruments X-Max 80 (Abingdon, Oxfordshire, UK) system. Typical detection limits in point analysis for different elements range between 0.3–0.5 at.%. The analytical data were normalized to 100 mas.%.

The oxygen content in some phases was determined by carbothermal reduction of oxide with subsequent analysis of the absorption of infrared radiation using an oxygen/hydrogen analyzer LECO-OH836 (St. Joseph, MI, USA). LECO Combustion Analysis can detect low level of oxygen with detection limit of 100 ppm and observed precision of 2.5%

### 2.5. Electrical Characterization

To measure the *ac* electrical conductivity, the cylindrical disc-shaped specimens were used. A thin layer of platinum paste was applied on both surfaces of each pellet and fired at the temperature of 900 °C for 1 h. Platinum wires were attached on the electrodes for measurements. The electrical conductivity measurements were carried out by a two-contact method using an impedance spectrometer (Z-1000P, Elins Ltd., Chernogolovka, Russia) over the temperature range of 300–1000 °C during cooling (1°/min) in air. The data were collected on cooling in 10–20 °C interval steps with the equilibrium time of 30 min. The electrical conductivity as a function of oxygen partial pressure (pO_2_ = 1 × 10^–18^ − 0.21 atm) was also measured from 500 to 900 °C. The oxygen partial pressure values were controlled and measured by 8 mol. % Y_2_O_3_-stabilized zirconia (YSZ)-sensor and by an YSZ-oxygen pump. Before the measurements, the impedance was monitored versus time to ensure that equilibrium was achieved. Typically, the equilibrium of the resistance for a given pO_2_ was achieved within 1–3 h for the pO_2_—range of 0.21 − 10^−5^ atm and within 5 h for the lower pO_2_-values.

To analyze the impedance spectra, the ZView-4.0 (Scribner Associate Inc., Southern Pines, NC, USA) program was used. The accuracy of impedance spectrometer was ±3%. To minimize the experimental error, the measurements were carried out on pellets with different geometries (thicknesses); each composition was tested for three times on different pellets. The measurement errors for a series of the samples did not exceed 10% for low temperatures (T < 500 °C) and 5% for higher temperatures.

The relative density of the ceramic samples was measured by Archimedes method in kerosene (ρ = 0.8190 g/cm^3^ at 20 °C). The bulk density of the sintered specimens was estimated from their mass and geometrical dimensions. The values of relative densities for investigated samples were found to be 98–99%. The ceramic samples of undoped phase Gd_2_Zr_2_O_7_ with the same relative density as the doped samples were prepared according to the scheme as described above, but at a higher temperature 1550 °C for 15 h. That is, as expected, MgO promoted the sintering densification [30,31]. The need to obtain high-density ceramics of Gd_2_Zr_2_O_7_ was due to the correct comparison of the electrical properties.

### 2.6. Chemical Stability Testing

Chemical stability was studied in a quartz cuvette in a glove box with an argon atmosphere (O_2_ < 2 ppm, H_2_O < 0.1 ppm). The LiCl (CAS No: 7447–41-8, Leverton Clarke Ltd., Basingstoke, UK) salt preliminarily purified by zone recrystallization together with annealed Li_2_O (99.5 mas.%, Alfa Aesar, Ward Hill, MA, USA) was loaded into a glassy carbon crucible. The concentration of lithium chloride after zone recrystallization was 99.92 mas.%.

The quartz cell was placed in a resistance furnace and heated to operating temperature. The temperature in the chamber was controlled using a thermostat; it was measured and maintained within ±2 °C using a platinum–platinum–rhodium thermocouple and a USB-TC01 thermocouple module (National Instruments, Austin, TX, USA). After the chloride mixture was melted, the ceramic sample was immersed and kept in the melt at the temperature of 650 °C. During the experiment, the melt was taken through the release of argon using quartz tubes to assess the degree of degradation of ceramics. After holding in the melt, the sample was removed, washed with distilled water and ethanol, dried at 100–150 °C for 2–3 h, subjected to isothermal distillation, and weighed. The sample was weighed before and after holding on a precision balance ±0.0001 g (OHAUS Corporation, Parsippany, NJ, USA).

The chemical composition of the melt was determined by the spectral emission method with inductively coupled plasma on an Optima 4300 DV optical emission spectrometer (Perkin Elmer, Waltham, MA, USA) with a detection limit of 0.0001−0.001 mas.% and an accuracy of 2 rel. %.

## 3. Results

### 3.1. Phase Characterization, Morphological and Elemental Analyses

**Figure 1:**
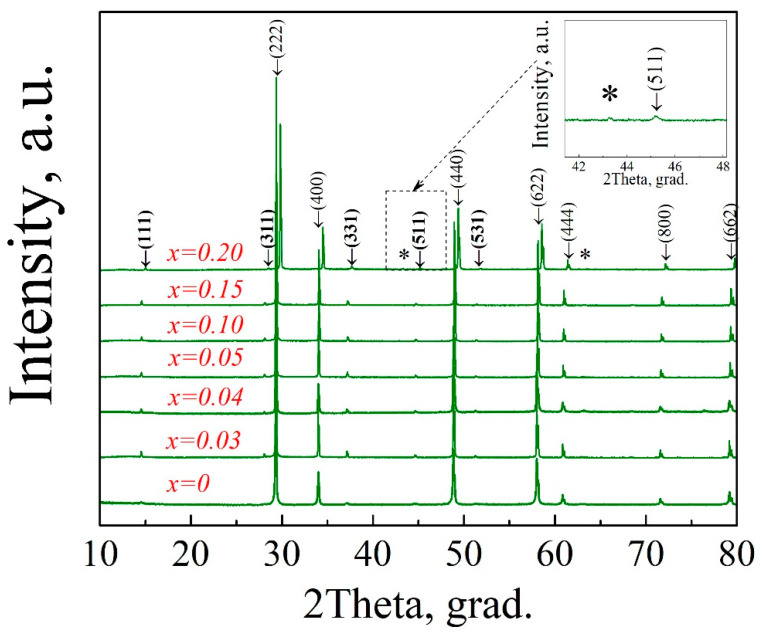
XRD patterns of Gd_2−x_Mg_x_Zr_2_O_7−x/2_ (x = 0, 0.03, 0.04, 0.05, 0.10, 0.15, 0.20). Numbers in parenthesis are the pyrochlore Miller indexes of each reflection (PDF Card No.: 01-080-7637). Superstructure pyrochlore Muller indexes are shown in bold. Magnesium oxide impurity is shown by asterisks (PDF Card No.: 01-075-0447). The insert shows an enlargement of some area of the XRD patterns.

Figure 1 shows X-ray diffraction patterns, obtained in air at T = 25 °C, for the samples Gd_2−x_Mg_x_Zr_2_O_7−x/2_ (x = 0; 0.03; 0.04; 0.05; 0.10; 0.15; 0.20). All obtained compositions 0 ≤ x ≤ 0.15 were isostructural with Gd_2_Zr_2_O_7_ and had a pyrochlore crystal structure (space group Fd-3m). The sample with the composition Gd_1.80_Mg_0.20_Zr_2_O_6.90_ had an MgO impurity in an amount of ~0.5% (the Rietveld refinement data). Although according to XRD data the sample with x = 0.15 was identified as single-phase, but investigation of microstructure by SEM showed the presence of the second phase MgO (the results will be presented below).

**Figure 2:**
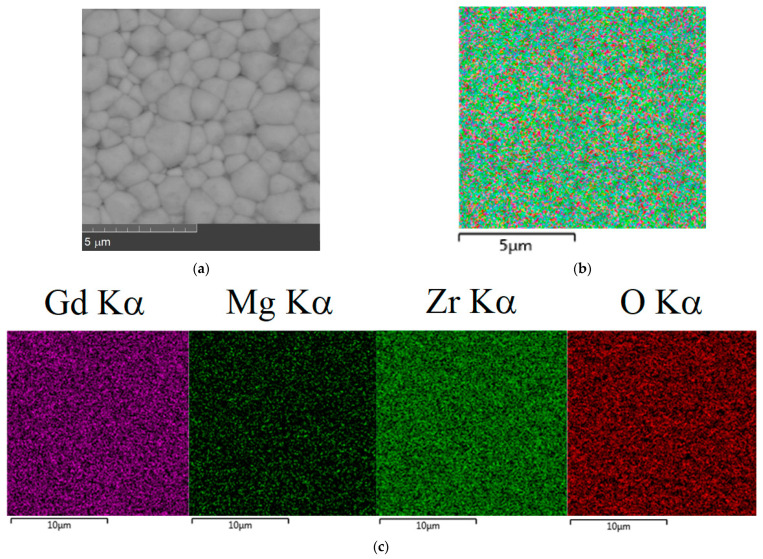
SEM images of Gd_2−x_Mg_x_Zr_2_O_7−x/2_ (x = 0.05) (**a**) and elemental mapping (**b**); gadolinium, magnesium, zirconium, and oxygen distribution for Gd_2−x_Mg_x_Zr_2_O_7−x/2_ (x = 0.05) (**c**).

**Figure 3:**
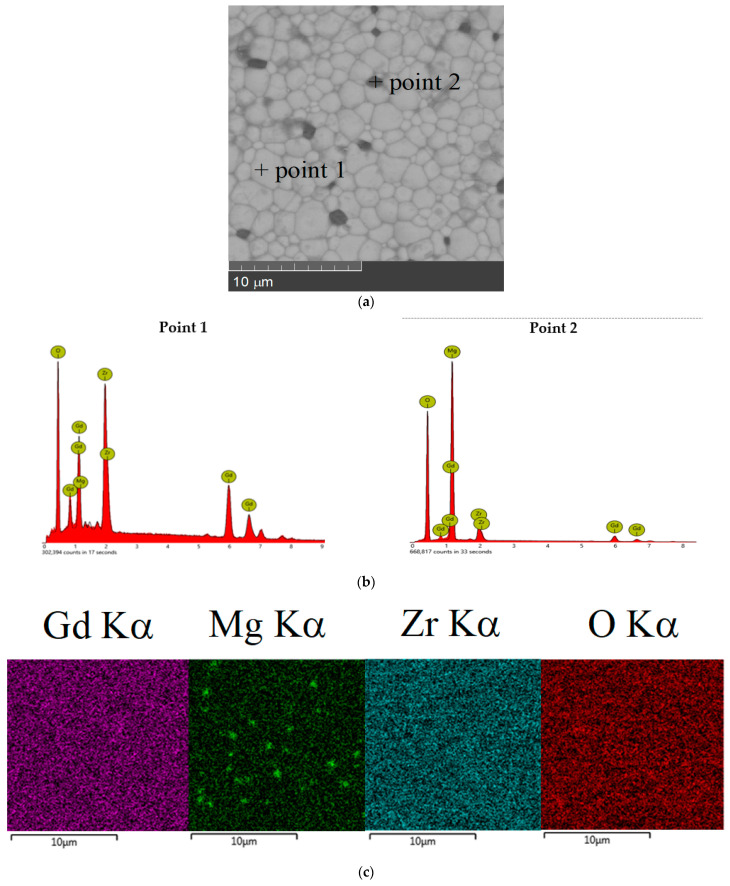
SEM images of Gd_2−x_Mg_x_Zr_2_O_7−x/2_ (x = 0.15) (**a**), EDS spectra for the points 1 and 2 (**b**); gadolinium, magnesium, zirconium, and oxygen distribution for Gd_2−x_Mg_x_Zr_2_O_7−x/2_ (x = 0.15) (**c**).

The SEM images showed that there are practically no pores in ceramics. Ceramics was composed of well-defined nearly uniform grains; agglomerations were not detected. Comparison of the surface morphology of the prepared materials revealed that the average grain size of the Mg^2+^-doped samples was about 1–5 µm and that there was no significant difference in surface morphology and particle size of the undoped and Mg-doped samples. To characterize the distribution of the chemical elements, FESEM-EDS with mapping analysis technique was used. Figure 2b,c and Figure 3c show typical EDS-mapping for two samples with x = 0.05 and x = 0.15 as examples of single-phase and non-single-phase samples. The single-phase samples with x ≤ 0.1 showed uniform distribution of the elements on the sample surface and indicated that Mg was homogeneously incorporated in the Gd_2_Zr_2_O_7_ lattice (Figure 2b,c). The quantitative EDS analysis confirmed that the chemical composition was in good agreement with theoretical chemical composition, as shown in Table 1. However, for the samples with x = 0.15 the distribution of the Mg was inhomogeneous (the same situation was for the sample with x = 0.20). As can be seen, the dark spots of the second phase clearly contrasted against the background of the main phase (Figure 3a). The EDS spectra obtained at different positions of 1 and 2 (Figure 3b) confirmed the presence of the pyrochlore type phase (point 1) and MgO phase (point 2). Obviously, low content of MgO impurity in the sample of x = 0.15 cannot be identified by XRD. So, the solubility limit of MgO in the solid solution Gd_2−x_Mg_x_Zr_2_O_7−x/2_ is near x = 0.15.

In order to characterize the elemental composition of the prepared samples, several methods have been used. Elemental analysis in SEM was performed using energy dispersive spectroscopy (EDS) and additional elemental analysis of the samples was carried out by atomic emission spectroscopy. Several samples were examined by the LECO oxygen measurement system to determine the oxygen content. The comparison of obtained data is shown in the Table 1. It was found that the mass ratios of the elements remained in good accordance with the theoretical compositions.

### 3.2. Structural Features

**Figure 4:**
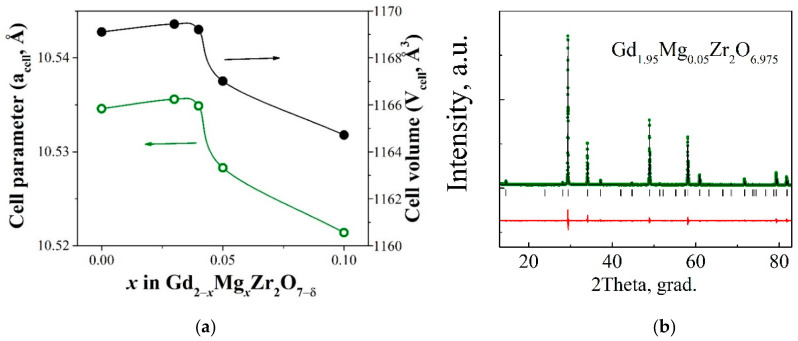
Concentration lattice parameter and cell volume dependencies for the solid solution Gd_2−x_Mg_x_Zr_2_O_7−x/2_ (**a**). Rietveld refinement XRD pattern of Gd_1.95_Mg_0.05_Zr_2_O_6.97_. Green dots are the experimental data, black line is the calculated data, black ticks are the peak positions of pyrochlore structure with space group Fd-3m, the red line is the difference between the calculated and the experimental data (R_p_ = 3.57, R_wp_ = 5.06, R_exp_ = 4.28, χ^2^ = 1.39) (**b**).

The dependence of the lattice parameter vs dopant concentration is shown in Figure 4a. As can be seen, there is a nonmonotonic dependence. The lattice parameter increases slightly for the composition x = 0.03 in comparison with undoped gadolinium zirconate [42] and then decreases monotonically. This suggests that different substitution mechanisms are realized depending on the concentration of the dopant. The ionic radii of Mg^2+^ and Gd^3+^ in eight-fold coordination are 0.89 Å and 1.053 Å, respectively [43]. Therefore, the substitution of Gd^3+^ in Gd_2_Zr_2_O_7_ with smaller Mg^2+^ should cause a decrease in the lattice parameter. However, we observe this trend only at higher dopant concentrations. At the same time, it should be said that the examples of the substitution of gadolinium for smaller ions in Gd_2_Zr_2_O_7_, accompanied by an increase in the lattice parameter, are known in the literature. For example, C. Wanga et al. [44] have reported that the lattice parameters of the solid solution (Gd_1−x_Sc_x_)_2_Zr_2_O_7_ increase with the increase in the Sc_2_O_3_ (R_Sc_ = 0.87 Å) content up to x = 0.075, at higher concentrations the lattice parameter decreases. Other compounds with the pyrochlore structure show the similar trends. For example, MgO-doped Sm_2_Zr_2_O_7_ [30] and Al_2_O_3_-doped Gd_2−y_Th_y_Zr_2_O_7_ [45] solid solutions exhibit initially increase of the lattice parameter with an increase in the doping content, and then decrease. The authors believe that the doping ions occupy of some interstitial sites of host pyrochlore structure and, consequently, increase in the lattice parameter occurs. Further decrease in the lattice parameter could be attributed to the substitution of host-ion sites with smaller dopant-ion. We believe that two substitution mechanisms are also possible for the investigated solid solution Gd_2−x_Mg_x_Zr_2_O_7−x/2_ and we observe the same dependences of the parameter vs the composition with a maximum as described in the literature. Figure 4b shows the X-ray pattern for the sample Gd_1.95_Mg_0.05_Zr_2_O_6.97_ refined by the Rietveld method as a typical example. Due to the high prominence of the superstructure lines (111), (311), (331), (511), and (531), a low degree of atomic inversion should be observed in the A- and B- sublattices of the doped samples (in other words, the mixing of the A and B sites decreases and the ordering increases).

It can be seen in Table 2 that the disorder of atoms decreased with increasing x in Gd_2−x_Mg_x_Zr_2_O_7−x/2_ for single-phase samples and ranged from 8.15 to 3.95%, respectively. The degree of disordering in the cationic positions in Gd_2_Zr_2_O_7_ reached 19%. Such structural feature is due to geometric criteria of the pyrochlore−fluorite transition. It is known that the ordered structure of pyrochlore is formed only at R_A_/R_B_ ≥ 1.46 [15] and when the radii of A and B cations in A_2_B_2_O_7_ are close, the ordering disappears. The phase Gd_2_Zr_2_O_7_ is located near the boundary of the pyrochlore−fluorite transition, accordingly the inversion of atoms occurs and 100% ordering is not achieved. It is interesting to note that the introduction of small magnesium into the A-sublattice leads to a decrease in zirconium content in the A-sublattice as a result of inversion. In this case, the content of gadolinium in the A-sublattice increases. So, upon doping average R_A_ radius increases (although overall the ratio R_A_/R_B_ is decreasing) and the mixing of Gd^3+^ and Zr^4+^ decreases.

It should be said that the results of this work are consistent with the data of A.V. Shlyakhtina et al. [33]. It was shown [33] that the XRD pattern of the Mg^2+^-doped composition Gd_1.9_Mg_0.1_Zr_2_O_6.95_ shows the well-defined (111), (311), (331), (511), and (531) superstructure reflections. That is, a high degree of ordering of the pyrochlore structure was observed during Mg^2+^-doping. On the other hand, when doping with calcium, that is with a larger cation than Gd^3+^ (RCa^2+^ = 1.12 Å, RGd^3+^ = 1.053 Å [43]), the opposite situation is observed: the superstructural lines disappeared. That is, disordering was observed during Ca^2+^-doping. That is, the size of the dopant-cation affects the degree of inversion of host-cations.

The disordering in the oxygen sublattice for the investigated solid solution is due to the formation of oxygen vacancies upon doping. In the framework of the quasi-chemical formalism the possible substitution reaction can be written as follows:

(1) $$2\mathrm{MgO}\overset{{\mathrm{Gd}}_{2}O_{3}}{\to }2{\mathrm{Mg}}_{\mathrm{Gd}}^{\prime }+2O_{o}^{x}+V_{o}^{\cdot \cdot }$$

where ${\mathrm{Mg}}_{\mathrm{Gd}}^{\prime }$ represents Mg^2+^-cation at a trivalent Gd-site, O_o_^×^ is oxygen on regular oxygen site, $V_{o}^{\cdot \cdot }$ is oxygen vacancy. It can be seen in Table 2, the introduction of Mg^2+^ in the A-sites was accompanied by the decrease in the occupancy of 48*f* site and the 8*b* site remains at the value of 0.56–0.60, given that the occupancy of 8*a* O(2) was constrained to be unity. This result is in a good agreement with the data obtained earlier and showing that a smaller energy is required to create a vacancy at 48*f* site O(1) [46,47].

Thus, with increasing Mg^2+^-content in the solid solution Gd_2−x_Mg_x_Zr_2_O_7−x/2_ the main disordering is associated with a decrease in the occupancy of the 48*f* oxygen positions, moreover, the inversion of Gd^3+^ and Zr^4+^ decreases.

### 3.3. Electrical Properties

**Figure 5:**
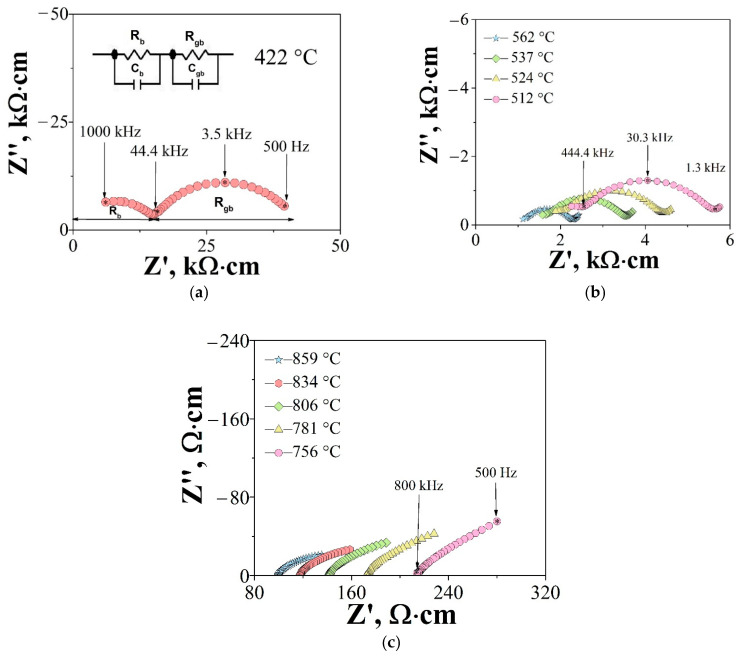
Typical *ac* impedance plot recorded at 422 °C as representation in the form of the Nyquist plot and corresponding equivalent circuit for Gd_1.97_Mg_0.03_Zr_2_O_6.98_ (**a**), at 512–562 °C (**b**), at 756–859 °C (**c**) in air. R_b_, R_gb_, CPE_b_, and CPE_gb_ represent bulk resistance, grain-boundary resistance, constant phase element of the bulk and constant phase element of the grain-boundary. Selected frequencies are indicated by arrows.

Figure 5a shows the typical impedance spectra for the Gd_1.97_Mg_0.03_Zr_2_O_6.98_ at T = 422 °C. In general, two impedance contributions could be identified at the temperatures T < 700 °C. The first arc in the high-frequency region started from zero, this contribution had a capacitance value of 10^−11^ F·cm^−1^ and was responsible for bulk properties of the material (R_b_). The second arc with the capacity value of 10^−9^ F·cm^−1^ described the grain boundaries contribution (R_gb_). With increasing temperature, the high-frequency part of the hodograph disappeared. Figure 5b shows a well-resolved arc in the temperature range of 512–562 °C, which described the grain boundaries contribution. The spectra were fitted using a simple equivalent circuit (R|CPE)(R|CPE). At the high temperature region T > 700 °C (Figure 5c) the spectra were characterized only by the electrode response. So, only the total resistance (R_b_ + R_gb_) can be determined for high temperature region. This evolution of hodographs is typical for ceramics based on Gd_2_Zr_2_O_7_ [33].

**Figure 6:**
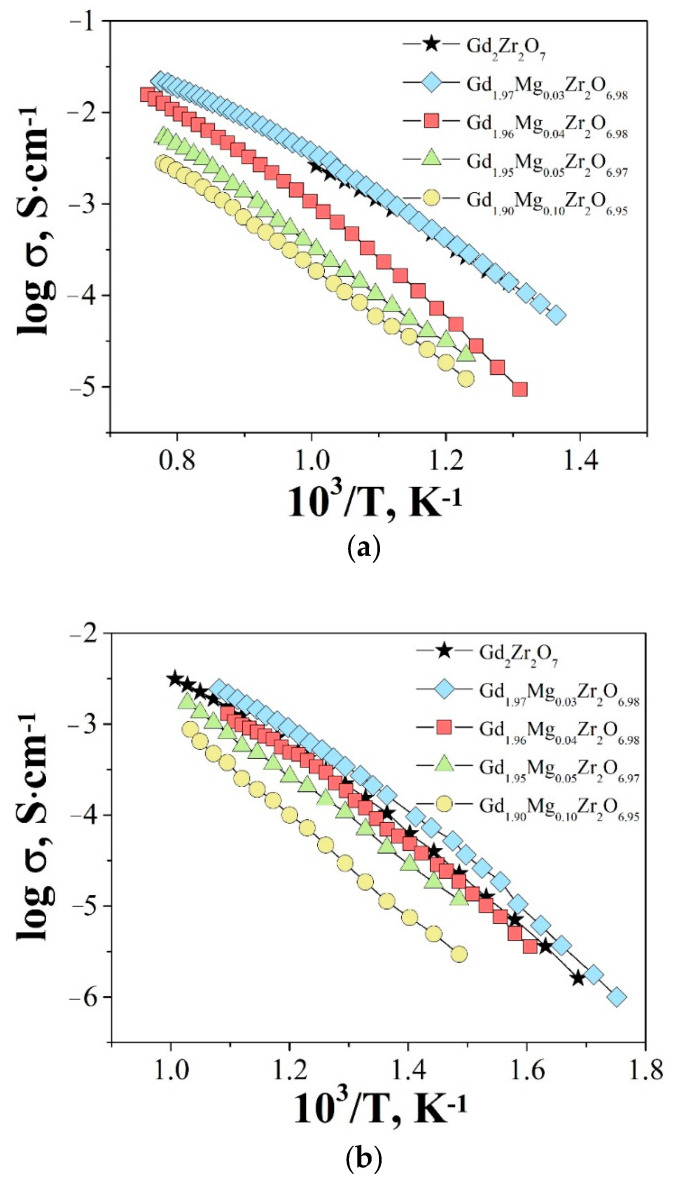
Temperature dependencies of total (**a**) and bulk (**b**) conductivities for Gd_2−x_Mg_x_Zr_2_O_7−x/2_ in air.

**Figure 7:**
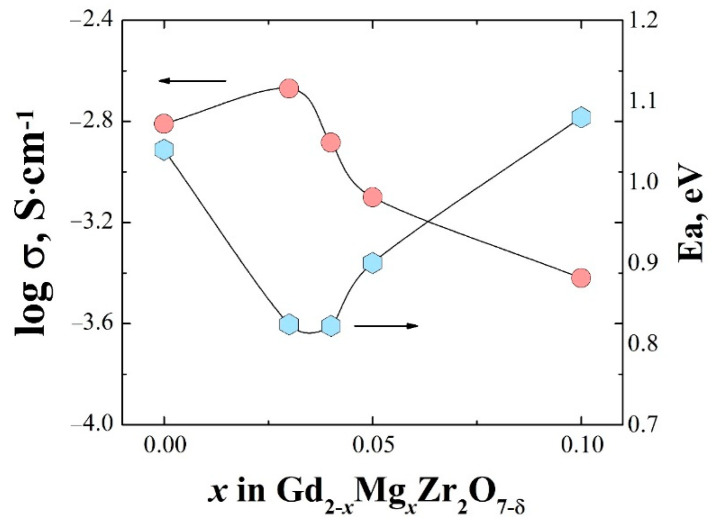
Concentration dependence of bulk conductivity and activation energy for Gd_2−x_Mg_x_Zr_2_O_7−x/2_ at 640 °C.

The temperature dependencies of total and bulk conductivities of the solid solution Gd_2−x_Mg_x_Zr_2_O_7−x/2_ are shown in Figure 6a,b. Both plots showed the same character depending on the concentration of the dopant: the Arrhenius plots of total and bulk conductivities of the composition Gd_1.97_Mg_0.03_Zr_2_O_6.98_ were higher than undoped Gd_2_Zr_2_O_7_. The concentration dependence of bulk conductivities and activation energies for Gd_2−x_Mg_x_Zr_2_O_7−x/2_ are shown in Figure 7. Within the solubility limit the bulk conductivity slightly increased at x = 0.03 and with further increasing dopant concentration conductivity decreased. As can be seen, the increase in electrical conductivity was accompanied by a decrease in the activation energy (Figure 7).

**Figure 8:**
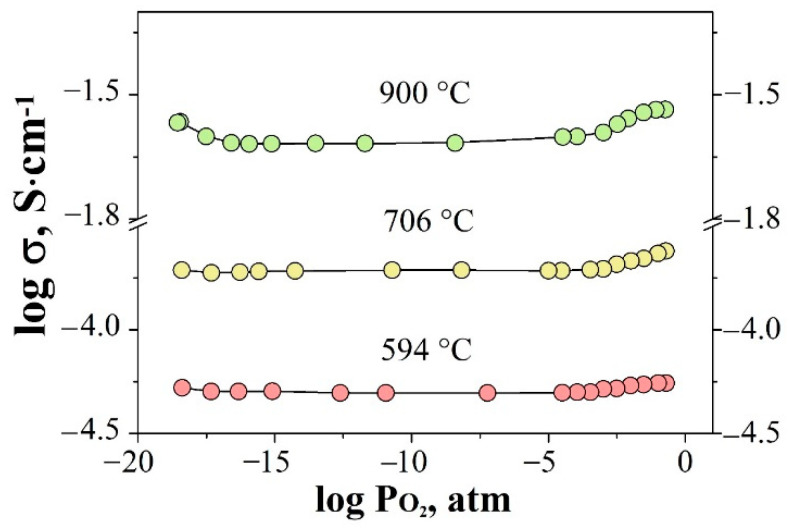
Oxygen partial pressure dependencies of conductivity for the sample Gd_1.95_Mg_0.05_Zr_2_O_6.97_ at different temperatures.

In order to establish the nature of the dominant type of carriers, the measurements of electrical conductivity as a function of oxygen partial pressure were performed. Figure 8 shows the results of measurements of conductivity vs pO_2_. The typical dependencies are shown for the composition Gd_1.95_Mg_0.05_Zr_2_O_6.97_. As can be seen, for the studied ranges of the temperatures and over a wide range of pO_2_, the sample showed independence of conductivity from pO_2_. At the high temperatures a slight negative slope was observed in the region of low pO_2_, indicating the presence of a small contribution of *n*-type conductivity, and also, at high pO_2_, a slight positive slope of the dependence was observed, which indicated the presence of a contribution from the *p*-type conductivity. These data show that all the investigated ceramics are characterized by the dominant ionic type of conductivity over a wide range of pO_2_ and at T < 800 °C.

Returning to the analysis of the concentration dependences of the conductivities (Figure 7), we can say that the dependencies reflect the behavior of the oxygen-ion conductivity. Only at very low concentrations (x = 0.03) a slight increase in the ionic conductivity was observed. To explain this behavior, one should consider the causes discussed in the literature.

As it is known, ionic conductivity in solid electrolytes is determined by the concentration of mobile current carriers *c_i_*, their charge *Z_i_e* and mobility *μ_i_*:

(2) $$\sigma_{i}=z_{i}\cdot e\cdot \mu_{i}\cdot c_{i}$$

The Mg^2+^-doping creates the additional number of oxygen vacancies. This is a favorable factor for the increase in oxygen-ion conductivity, but we can observe this trend only in the region of low dopant concentrations (x ≤ 0.03). With a further increase in the concentration of the dopant, the ionic conductivity decreases. This shows the influence of another factor with the opposite effect. Obviously, this is due to a decrease in the mobility. In the most general case, the mobility is determined by the binding energy of the mobile ion with the crystal lattice and the geometry of the unit cell. At the same time, the specific structure of pyrochlore suggests the need to analyze other factors caused by a change in the degree of disorder of cations.

It is necessary to consider explanations in the literature for the concentration behavior of oxygen-ion conductivity in doped gadolinium zirconate Gd_2_Zr_2_O_7_. For example, for the solid solution GdSm_1−x_Mg_x_Zr_2_O_7−x/2_ it was expected to improve the conduction through creation of oxygen vacancies, but on the contrary, during doping the activation energy and pre-exponential factor increased, and total conductivity decreased [31]. That is, the increase in the pre-exponential factor was not compensate the rise in activation energy, and thus the ionic conductivity decreased. Ca^2+^-doping of GdSmZr_2_O_7_ [48] and Gd_2_Zr_2_O_7_ [33] also did not lead to an increase in conductivity. For Li^+^-doped solid solution Gd_2−x_Li_x_Zr_2_O_7−x_, it was shown that although the lattice parameter decreased with doping, the free volume increased [49]. This led to an increase in ionic conductivity at low dopant concentrations up to one order of magnitude.

At the same time, isovalent doping can lead to an increase in ionic conductivity, as it was shown for the solid solutions Gd_2−x_Nd_x_Zr_2_O_7_ [23] and Gd_2−x_La_x_Zr_2_O_7_ [50]. Activation energy decreased with increase in the Nd^3+^ and La^3+^-content and this is a favorable factor for increasing conductivity. This doping led to an increase in the R_A_/R_B_ ratio and was accompanied by increase in ordering. The authors explain [50] that the increase in conductivity upon La^3+^-doping is the result of the weakening of ion–ion interactions, promoted by the more ordered structure, consequently, contributed to an increase in the mobility of ions.

These results indicate that there is no common understanding of the factors affecting oxygen mobility in pyrochlores, but at the same time, it is clear that geometrical factors affect oxygen mobility.

Returning to the investigated system Gd_2−x_Mg_x_Zr_2_O_7−x/2_, we can assume that the increase in oxygen-ion conductivity is determined not only by an increase in the concentration of vacancies, but also due to the expansion of the lattice in the region of low concentrations of Mg^2+^ (x ≤ 0.03). The increase in conductivity is accompanied by a decrease in the activation energy as a result of weakening the metal–oxygen bonding due to the cell volume expansion. A significant decrease in the lattice parameter with a further increase in the dopant concentration leads to a drop of conductivity. As can be seen from the data in Table 3, pre-exponential factor in the σT = A·exp(−E*_a_*/kT) (σ is the electrical conductivity, T is the absolute temperature, E*_a_* is the activation energy, k is the Boltzmann constant) decreases with increasing magnesium oxide concentration, i.e., with a decrease in the lattice parameter. This is consistent with the reasoning presented above.

Summarizing the presented results, we can conclude that although the concentration of defects is a parameter that determines the behavior of ionic conductivity, but the second parameter is the ion mobility, which changes more significantly in pyrochlores depending on the factors discussed above.

### 3.4. Chemical Stability in the Melt of LiCl-Li_2_O

The chemical stability of oxide materials is an important parameter to evaluate their ability for device applications. One such important application is the development in the field of sensor materials. In this regard, the study of the chemical stability of complex oxides in halide melts is an important task. Ceramic samples of the compositions x = 0.05 and 0.10 were investigated in terms of their stability in the melt of LiCl–Li_2_O with different concentrations of lithium oxide.

**Figure 9:**
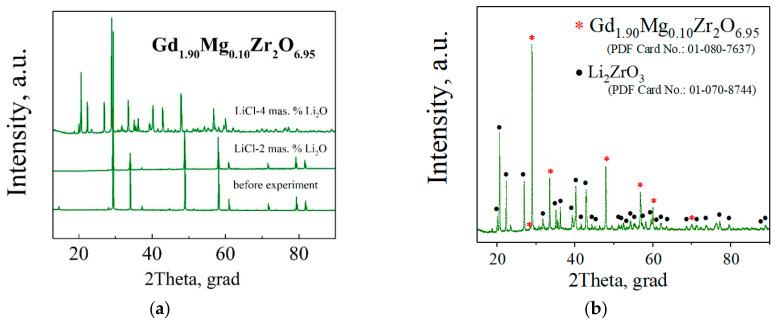
XRD patterns of the sample Gd_1.90_Mg_0.10_Zr_2_O_6.95_ before the melt holding experiment (PDF Card No.: 01-080-7637), exposed for 52 h in the melt of LiCl–Li_2_O (2 mas.%) at 650 °C, exposed for 48 h in the melt of LiCl–Li_2_O (4 mas.%) at 650 °C (**a**). XRD pattern of the sample Gd_1.90_Mg_0.10_Zr_2_O_6.95_, exposed for 48 h in the melt of LiCl–Li_2_O (4 mas.%) (**b**).

According to the data of X-ray phase analysis (Figure 9a,b), after holding ceramics in the melt of LiCl with 2 mas.% Li_2_O additives at 650 °C, the samples remained single-phase during the experiment time of one week.

However, after holding the samples for 48 h in the melt of LiCl with 4 mas.% Li_2_O at 650 °C, an impurity phase Li_2_ZrO_3_ was found on the ceramic surface, but the ceramics did not crack. Chemical analysis (Table 4) of the elemental composition of the melt did not reveal the transition of the solid phase components (Gd, Mg, Zr) into the melt. The presence of silicon in the melt is due to the contact of the melt with a quartz tube for the melt sampling.

**Figure 10:**
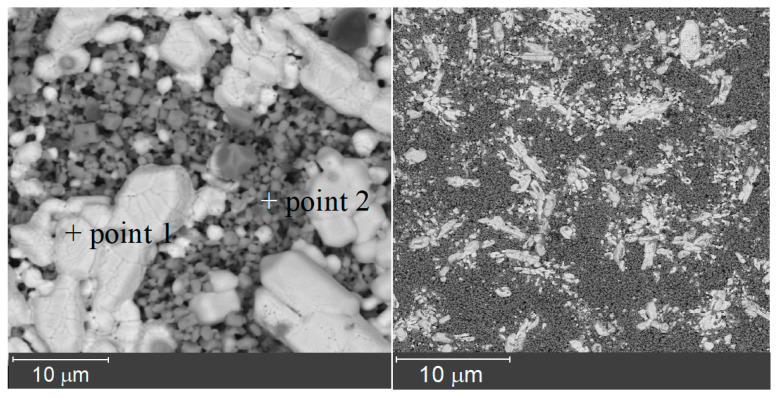
SEM images for the ceramic sample of Gd_1.90_Mg_0.10_Zr_2_O_6.95_, exposed for 48 h in LiCl–Li_2_O (4 mas.%) melt.

Microstructure of the surface of the samples after their interaction with the LiCl-4 mas.% Li_2_O melt is shown in Figure 10. The appearance of new phases is clearly seen from the different morphology and contrast of the ceramic surface. The lighter areas (point 1; O-71.25 at.%, Zr-5.70 at.%, Gd-23.05 at.%) correspond to areas with an increased concentration of gadolinium, and the dark areas (point 2; O-84.65 at.%; Zr-14.01 at.%; Gd-1.13 at.%) correspond to zirconium enrichment.

Thus, it can be concluded that the solid solution Gd_2−x_Mg_x_Zr_2_O_7−x/2_ can be used for electrochemical applications in the LiCl melt with a lithium oxide content less than 4 mas.%.

## 4. Conclusions

Mg^2+^-substituted pyrochlores Gd_2−x_Mg_x_Zr_2_O_7−x/2_ (x ≤ 0.10) were prepared by mechanosynthesis technique. Sintering at 1500 °C yielded dense ceramics with relative density 98–99%. FE-SEM/EDX investigations indicated homogeneity of cation distribution with average cation composition very close to the nominal composition. Refinement of XRD data revealed an increase in lattice parameter for the small concentration (x = 0.03), although the substitution of gadolinium for smaller Mg^2+^ ions should have the opposite effect. We assume that at low dopant concentrations some interstitial positions can be substituted by Mg^2+^. With further increasing Mg^2+^—content, the decrease in lattice parameter occurred. All studied Gd_2−x_Mg_x_Zr_2_O_7−x/2_ ceramics were ionic conductors in air (T < 800 °C). The behavior of oxygen-ion conductivity depending on the dopant concentration was characterized by a small maximum. The sample with the composition of x = 0.03 had the highest oxygen-ion conductivity (10^−3^ S·cm^−1^ at 600 °C). The investigation of chemical stability showed that the samples were chemically stable in the melts of LiCl–Li_2_O with concentration of lithium oxide of not more than 2 mas.% and this makes it possible to use these materials as oxygen activity sensors in the halide melts.

## Figures and Tables

**Table 1:** Elemental composition, determined by the EDS and chemical analyzes, and oxygen content determined by the LECO analysis, for the investigated samples.

Samples	Theoretical Values, mas.%				EDS Analysis, mas.%				Leco, mas.%	Chemical Analysis, mas.%			
	Gd	Mg	Zr	O	Gd	Mg	Zr	O	O	Gd	Mg	Zr	O
Gd_2_Zr_2_O_7_	51.65	-	29.96	18.39	50.93	-	30.01	19.06			-		
Gd_1.97_Mg_0.03_Zr_2_O_6.98_	51.23	0.12	30.17	18.48	51.57	0.19	30.08	18.16	-				
Gd_1.96_Mg_0.04_Zr_2_O_6.98_	51.09	0.16	30.24	18.51	51.04	0.23	30.38	18.35	-				
Gd_1.95_Mg_0.05_Zr_2_O_6.97_	50.95	0.20	30.31	18.54	50.84	0.30	29.96	18.90	18.50	49.20	0.26	28.80	21.74
Gd_1.90_Mg_0.10_Zr_2_O_6.95_	50.23	0.41	30.67	18.69	49.87	0.52	30.98	18.63	19.80	48.80	0.44	28.40	22.36

**Table 2:** The site occupancies for Gd_2−x_Mg_x_Zr_2_O_7−x/2_ in a cubic unit cells with the space group Fd-3m.

Composition	Site Occupancies							
	Gd	Mg	Zr	Zr	Gd	O	O	O
	16c	16c	16c	16d	16d	48f	8a	8b
Gd_2_Zr_2_O_7_	1.809	-	0.191	1.809	0.191	5.849	1.000	0.151
Gd_1.97_Mg_0.03_Zr_2_O_6.98_ *	1.807	0.030	0.163	1.837	0.163	5.424	1.000	0.560
Gd_1.96_Mg_0.04_Zr_2_O_6.98_	1.809	0.040	0.151	1.849	0.151	5.365	1.000	0.615
Gd_1.95_Mg_0.05_Zr_2_O_6.97_	1.819	0.050	0.131	1.869	0.131	5.355	1.000	0.615
Gd_1.90_Mg_0.10_Zr_2_O_6.95_	1.821	0.100	0.079	1.921	0.079	5.351	1.000	0.599

* The Rietveld treatment of the composition x = 0.03 was carried out in accordance with the presented formula of the solid solution, since it is impossible to determine the coordinates for magnesium due to its very low concentration.

**Table 3:** Oxygen ion conductivity values (σ) at 640 °C, activation energies (E*_a_*) and pre-exponential factor (A) for Gd_2−__x_Mg_x_Zr_2_O_7−x/2_.

x	−log σ (S·cm^−1^)	E_*a*_ (eV) ± 0.01	A (S·cm^−1^ K) × 10^−5^
0.00	2.72	1.11	25.06
0.03	2.66	0.82	5.51
0.04	2.87	0.82	3.65
0.05	3.10	0.90	0.77
0.10	3.42	1.08	0.04

**Table 4:** Chemical analysis of the LiCl-4 mas.% Li_2_O melt after 48 h holding the samples. Gd_2−x_Mg_x_Zr_2_O_7−x/2_ (x = 0.05, 0.1).

Analyzed Element	Gd	Mg	Zr	Si	Al
Concentration, mas.%	<0.0001	<0.001	<0.0001	0.049	<0.001
